# 386. Pathogen-Harboring Ticks are Prevalent in Suburban Green Spaces in Central North Carolina

**DOI:** 10.1093/ofid/ofae631.121

**Published:** 2025-01-29

**Authors:** Abigail Schulz, Jack Dalton, Haley Abernathy, Lanya Evans, Johnathan Hicks, Adjaratou Diouf, Dana Giandomenico, Ross M Boyce

**Affiliations:** University of Illinois College of Medicine at Peoria, Peoria, IL; University of Leeds, Leeds, England, United Kingdom; Institute for Global Health and Infectious Diseases, University of North Carolina, Chapel Hill, North Carolina; University of North Carolina at Chapel Hill, Chapel Hill, North Carolina; North Carolina State University, Raleigh, North Carolina; North Carolina State University, Raleigh, North Carolina; University of North Carolina at Chapel Hill, Chapel Hill, North Carolina; University of North Carolina at Chapel Hill, Chapel Hill, North Carolina

## Abstract

**Background:**

North Carolina (NC) reports high numbers of ehrlichiosis and spotted fever group rickettsioses. However, the prevalence of pathogens in ticks has historically been relatively low, with a < 10% prevalence of *Ehrlichia* spp. and < 1% prevalence of *Rickettsia rickettsii* in field-collected *Amblyomma americanum* and *Dermacentor variabilis* ticks. Most entomological surveys have focused on woodlands and rural sites, while less is known about the risk of human-tick interactions in suburban green spaces such as parks.

**Table 1.**
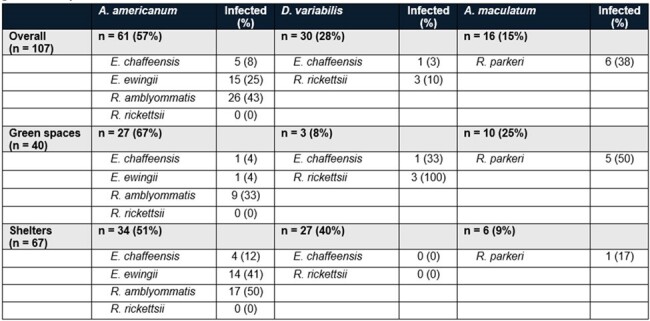
Pathogens identified in ticks in central North Carolina from surveillance in suburban green spaces and animal shelters.

**Methods:**

The objective of the study, which was supported by an IDSA GERM Award, was to describe the prevalence and distribution of pathogen-harboring ticks in a suburban area of central NC with a focus on green spaces. Tick surveillance was performed by dragging vegetation in 16 suburban green spaces, primarily city and county parks, in Orange and Chatham Counties from June - August 2022. Ticks were also collected from stray dogs presenting to two county animal shelters. Polymerase chain reaction (PCR) was used to detect pathogens in the ticks.

**Results:**

A total of 107 ticks were collected and underwent PCR testing (**Table 1**), including 40 collected via dragging and 67 from animal shelters. *A. americanum* were the most frequently encountered ticks, accounting for more than two-thirds of ticks collected in green spaces and half of ticks from shelters. A total of 26% were positive for *E. chaffeensis* or *E. ewingii*, with substantially higher infection rates among ticks from shelters. *A. maculatum* accounted for 25% of ticks in green spaces, half of which were positive for *R. parkeri*. The vast majority (90%) of *D. variabilis* ticks were collected from shelters, but all three ticks from green spaces were positive for *R. rickettsii*. While of uncertain pathogenicity, *R. amblyommatis* was the most frequently encountered bacterium among all ticks collected.

**Conclusion:**

Our findings demonstrate that pathogen-infected ticks are present in suburban green spaces. Given that exposure risk of tick-borne disease is commonly associated with rural activities such as hiking, camping, and hunting, further education and public campaigns are warranted, and vector control efforts may need to be considered. Our findings also suggest that *A. maculatum* may be moving further inland than previously reported.

**Disclosures:**

**All Authors**: No reported disclosures

